# Normoxic Management during Cardiopulmonary Bypass Does Not Reduce Cerebral Mitochondrial Dysfunction in Neonatal Swine

**DOI:** 10.3390/ijms25105466

**Published:** 2024-05-17

**Authors:** Danielle I. Aronowitz, Tracy R. Geoffrion, Sarah Piel, Sarah R. Morton, Jonathan Starr, Richard W. Melchior, Hunter A. Gaudio, Rinat Degani, Nicholas J. Widmann, M. Katie Weeks, Nicolina R. Ranieri, Emilie Benson, Tiffany S. Ko, Daniel J. Licht, Marco Hefti, J. William Gaynor, Todd J. Kilbaugh, Constantine D. Mavroudis

**Affiliations:** 1 Division of Cardiothoracic Surgery, Children’s Hospital of Philadelphia, Philadelphia, PA 19104, USA; daniarono@gmail.com (D.I.A.);; 2 Resuscitation Science Center of Emphasis, Children’s Hospital of Philadelphia, Philadelphia, PA 19104, USA; 3 Department of Neurology, Children’s Hospital of Philadelphia, Philadelphia, PA 19104, USA; 4 Department of Physics & Astronomy, University of Pennsylvania, Philadelphia, PA 19104, USA; 5 Department of Pathology, University of Iowa Health Care, Iowa City, IA 52242, USA

**Keywords:** cardiopulmonary bypass, animal models, brain injury

## Abstract

Optimal oxygen management during pediatric cardiopulmonary bypass (CPB) is unknown. We previously demonstrated an increase in cortical mitochondrial reactive oxygen species and decreased mitochondrial function after CPB using hyperoxic oxygen management. This study investigates whether controlled oxygenation (normoxia) during CPB reduces cortical mitochondrial dysfunction and oxidative injury. Ten neonatal swine underwent three hours of continuous CPB at 34 °C (flow > 100 mL/kg/min) via cervical cannulation targeting a partial pressure of arterial oxygen (PaO_2_) goal < 150 mmHg (normoxia, n = 5) or >300 mmHg (hyperoxia, n = 5). The animals underwent continuous hemodynamic monitoring and serial arterial blood sampling. Cortical microdialysate was serially sampled to quantify the glycerol concentration (represents neuronal injury) and lactate-to-pyruvate ratio (represents bioenergetic dysfunction). The cortical tissue was analyzed via high-resolution respirometry to quantify mitochondrial oxygen consumption and reactive oxygen species generation, and cortical oxidized protein carbonyl concentrations were quantified to assess for oxidative damage. Serum PaO_2_ was higher in hyperoxia animals throughout CPB (*p* < 0.001). There were no differences in cortical glycerol concentration between groups (*p* > 0.2). The cortical lactate-to-pyruvate ratio was modestly elevated in hyperoxia animals (*p* < 0.03) but the values were not clinically significant (<30). There were no differences in cortical mitochondrial respiration (*p* = 0.48), protein carbonyls (*p* = 0.74), or reactive oxygen species generation (*p* = 0.93) between groups. Controlled oxygenation during CPB does not significantly affect cortical mitochondrial function or oxidative injury in the acute setting. Further evaluation of the short and long-term effects of oxygen level titration during pediatric CPB on cortical tissue and other at-risk brain regions are needed, especially in the presence of cyanosis.

## 1. Introduction

There are no evidence-based guidelines for optimal oxygen management during pediatric cardiopulmonary bypass (CPB). Hyperoxic oxygen management is typically used during CPB to ensure adequate systemic oxygen delivery, which may be limited by hemodilution and non-pulsatile flow during extracorporeal circulation. This practice has recently been called into question due to the potential adverse effects of supranormal oxygen levels and subsequent oxidative stress [1,2,3]. We previously demonstrated increased cerebral reactive oxygen species (ROS) and decreased mitochondrial function in neonatal swine treated with “standard” CPB using hyperoxia (partial pressure of oxygen, PaO_2_ > 250 mmHg) [4,5], and pediatric swine exposed to hyperoxia (100% inspired oxygen) during cardiopulmonary resuscitation after cardiac arrest [6]. Mitochondria regulate energy generation, oxygen metabolism, and free radical homeostasis in the cell. Pathophysiologic states of hyperoxia or hypoxia can stress and damage mitochondrial function. When mitochondrial dysfunction reaches a critical point, cellular bioenergetic imbalance causes irreversible cellular injury, cell death, and propagates further neuroinflammatory cascades [7]. As such, mitochondrial dysfunction represents a critical target for both diagnostics and therapeutics to mitigate downstream cellular death in response to stressors.

This study investigates whether controlled oxygenation (normoxia) during CPB decreases oxidative injury to cerebral mitochondria and downstream cellular injury.

## 2. Results

### 2.1. Animal Characteristics at Baseline and during CPB

The animal characteristics at baseline are summarized in Table 1. There were no differences in animal characteristics, hemodynamic parameters, or blood gas measurements between the normoxia and hyperoxia groups at baseline (Table 1). Successful implementation of controlled oxygenation throughout CPB in the normoxia group resulted in a significantly higher PaO_2_ in the hyperoxia group throughout CPB (*p* < 0.01, Table 2 and Table 3). The mean hematocrit was lower in the normoxia group in the second hour of CPB (normoxia 32.8% vs. hyperoxia 36.2%, *p* = 0.02, Table 3). In the third hour of CPB, the heart rate (hyperoxia 117 bpm vs. normoxia 157 bpm, *p* = 0.02) and systolic blood pressure (hyperoxia 61 mmHg vs. normoxia 76.7 mmHg, *p* = 0.01) were lower in the hyperoxia group. There were no other differences in arterial blood gas or hemodynamic measurements between the two groups (Table 3).

### 2.2. Cerebral Microdialysis

**Figure 1:**
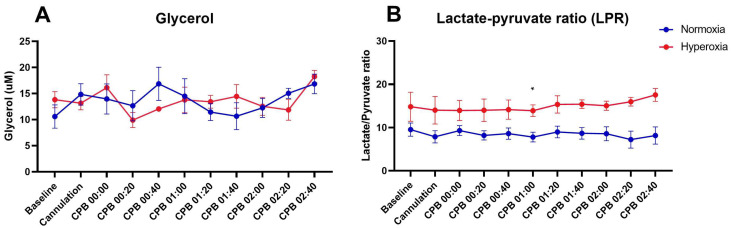
Cerebral microdialysis. There was no difference in the mean concentration of glycerol at baseline or at any timepoint during CPB between groups ((**A**), *p* > 0.7 at each time point). The mean LPR was slightly higher in the hyperoxia group compared with the normoxia group throughout the experiment, but this trend only reached significance at 1 h of CPB ((**B**), * *p* < 0.01). Furthermore, the LPR values for all time points in both groups were within the normal range (17–27) as reported in human studies. The values are plotted as mean and standard error of the mean. The means were compared by two-way ANOVA with Sidak multiple comparisons test.

Increasing glycerol levels in cerebral microdialysate indicate brain tissue injury [8]. A rising lactate-to-pyruvate ratio indicates a shift toward anaerobic glycolysis. Ratio values of 17–27 have been previously reported as normal [9,10] and values > 30 have been associated with poor outcomes in human studies [11,12]. There was no difference between groups in the concentration of glycerol in cerebral interstitial fluid at baseline or during CPB (*p* > 0.7, Figure 1A). As shown in Figure 1B, mean LPR was slightly higher in the hyperoxia group compared with the normoxia group at baseline and throughout the experiment, but this trend only reached significance at 1 h of CPB (*p* < 0.01, Figure 1B). That being said, LPR in the hyperoxia group never exceeded 30, which is generally the threshold of negative clinical outcomes [11,12].

### 2.3. Markers of Cortical Mitochondrial Function, Content, and Oxidative Stress

**Figure 2:**
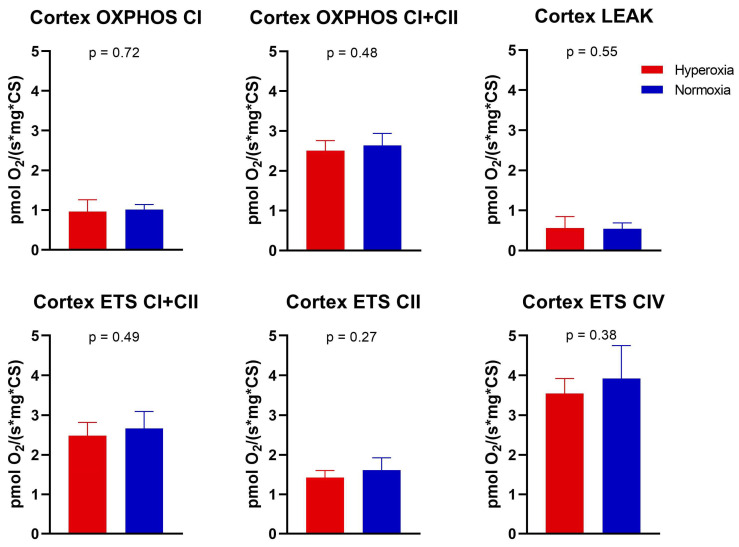
Mitochondrial respiration. Oxygen consumption rates for the six respiratory states measured by high-resolution respirometry. Values were normalized to the citrate synthase activity. There was no difference between the hyperoxia and normoxia groups for any state. Values are plotted as mean and standard error of the mean. The means were compared by one-way ANOVA or Kruskal−Wallis test.

**Figure 3:**
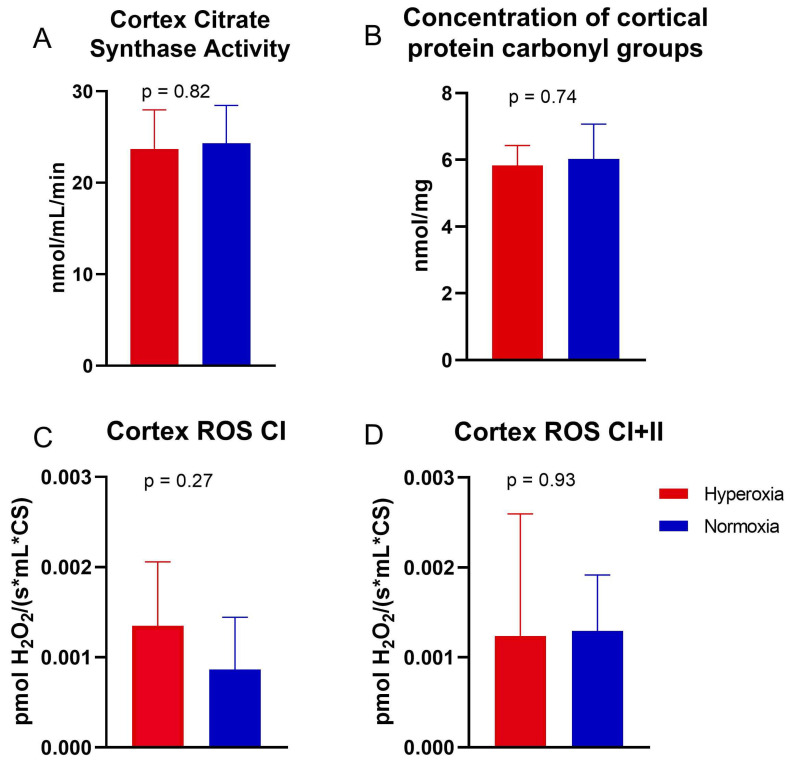
Citrate synthase activity. (**A**) Mean citrate synthase activity (as a surrogate of mitochondrial content) in the cerebral cortex. There was no significant difference in the mean citrate synthase activity between the two groups. (**B**) The mean concentration of oxidized cortical protein carbonyl groups was not significantly different between the two groups. The cortical reactive oxygen species (ROS) levels did not differ between the groups at (**C**) OXPHOS CI and (**D**) OXPHOS CI+CII. The values are plotted as the mean and standard error of the mean. Means were compared by one-way ANOVA or Kruskal−Wallis test.

The following respiratory states of the electron transport chain were measured: maximal oxidative phosphorylation via complex I (OXPHOS CI), maximal convergent oxidative phosphorylation via complexes I and II (OXPHOS CI+CII), oligomycin-induced proton cycling through mitochondria without ATP synthesis (LEAK), maximal convergent non-phosphorylating respiration of the electron transport system via complexes I and II (ETS CI+CII), and via complex II alone (ETS CII), and complex IV activity. The cortical mitochondrial respiration via each respiratory state tested was not different between the two groups (Figure 2). There was no difference in OXPHOS CI (*p* = 0.72), OXPHOS CI+CII (*p* = 0.48), LEAK (*p* = 0.55), ETS CI+CII (*p* = 0.49), ETS CII (*p* = 0.27), or complex IV activity (*p* = 0.38). There was no difference in citrate synthase activity (*p* = 0.82, Figure 3A), or the concentration of oxidized cortical protein carbonyl groups (*p* = 0.74, Figure 3B) between groups. The cortical ROS levels at OXPHOS CI and OXPHOS CI+CII also did not differ between groups (*p* = 0.27 and 0.93, respectively, Figure 3C,D).

## 3. Discussion

We found that controlled oxygenation (normoxia, PaO_2_ < 150 mmHg) during CPB does not affect cortical mitochondrial function, downstream indicators of oxidative injury, or bioenergetic failure compared to standard oxygenation (hyperoxia, PaO_2_ > 300 mmHg). The lactate-to-pyruvate ratio in the cerebral interstitial fluid increased slightly in the hyperoxia group in the second two-thirds of CPB. This may reflect a minor shift toward increased lactate production and/or reduced pyruvate consumption in the presence of hyperoxia. However, this finding likely has no clinical significance as the mean LPR did not exceed the threshold at which negative clinical outcomes would be expected in either group. There was no difference in the concentration of glycerol in the cerebral interstitial fluid or cortical protein carbonyl groups between the two groups to suggest brain tissue injury or oxidative damage. These findings suggest that normoxic management during CPB does not prevent mitochondrial injury, bioenergetic dysfunction, or oxidative stress in the acyanotic brain.

The optimal oxygen management strategy during pediatric CPB remains unclear; recent clinical studies examining the impact of hyperoxic CPB on perioperative outcomes have yielded conflicting results. In an observational study, Asaad et al. evaluated the association between intraoperative PaO_2_ and 30-day mortality in infants (median age = 97 days, interquartile range = 14–179 days) undergoing cardiac surgery for either cyanotic or acyanotic congenital heart disease [3]. The authors found that hyperoxia (PaO_2_ > 313 mmHg) during CPB resulted in four-fold greater odds of 30-day mortality. In a separate study, Caputo et al. conducted two randomized controlled trials comparing the use of hyperoxic CPB (PaO_2_ between 150–200 mmHg) versus CPB with controlled oxygenation where the patient’s preoperative arterial oxygen saturation was maintained; compared with Asaad et al., the patients were relatively older (median age within interventional groups ranged from 10.4–14.7 months) and had exclusively cyanotic congenital heart disease [13]. In the combined cohort, they did not find any clinical advantage of controlled oxygenation during CPB over the more standard hyperoxic strategy.

Similarly, our study did not find any protective effect of controlled oxygenation during CPB on cortical mitochondrial function or oxidative stress compared to hyperoxic CPB. However, there are several factors to consider when generalizing the results of our oxidative stress analysis. Our analysis was conducted in an acyanotic animal model; generalization to cyanotic congenital heart disease may be limited as ROS generation and decreased mitochondrial function may be more detrimental in the presence of cyanosis where the cerebral bioenergetic reserve is preemptively limited. We planned to expand upon these data by including a cyanotic animal model of CPB in future work. Additionally, all animals underwent systemic cooling to mild hypothermia, but none of the animals underwent rewarming before necropsy and tissue harvest. The absence of a rewarming period could limit the clinical application of this animal model, but we do not believe this impacted our isolated evaluation of oxygen management during CPB. This study was also limited by a small sample size, isolation to the CPB period, and only neurological outcome measures. The reversibility of oxidative stress after separation from hyperoxic CPB and the effects of hyperoxic CPB on other organ systems remain significant knowledge gaps and require additional experiments using both acyanotic and cyanotic animal models.

## 4. Materials and Methods

### 4.1. Experimental Design

The study utilized a previously described neonatal swine model of CPB [4,5,14,15]. The animal care and procedures were approved by the Institutional Animal Care and Use Committee in accordance with the National Institutes of Health Guide for the Care and Use of Laboratory Animals.

Ten neonatal female Yorkshire piglets underwent 3 h of CPB (flow rate > 100 mL/kg/min) at 34 °C using either standard hyperoxic oxygenation (n = 5, PaO_2_ > 300 mmHg) or controlled normoxic oxygenation (n = 5, PaO_2_ < 150 mmHg). The perioperative preparation, CPB circuitry, priming, and monitoring methods are described elsewhere [4,5,14,15]. Detailed descriptions of the operative technique, tissue processing, and molecular assays are provided in the Supplementary Materials. 

### 4.2. Outcome Measures

Cerebral microdialysate (i.e., interstitial fluid representative of cerebral extracellular fluid) was collected at baseline and at 20-min intervals during CPB. The samples were analyzed for glycerol, lactate, and pyruvate levels [4,5,6,12]. The raw values at each sampling timepoint were summarized by the mean and standard error of the mean across all animals for each oxygenation group. Within each group, the mean values for every timepoint were then compared to the mean value at the end of CPB by one-way ANOVA.

Following euthanasia, fresh brain tissue samples were prepared and analyzed by high-resolution mitochondrial respirometry and normalized to citrate synthase activity, as in previous reports [4,5,6,12]. Citrate synthase is a mitochondrial enzyme used as a surrogate of mitochondrial content [12]. The citrate synthase activity (nmol/mg/min) was measured in cortical homogenates using a commercialized kit (Citrate Synthase Assay Kit, CS0720, Sigma-Aldrich, St. Louis, MO, USA) according to the manufacturer’s instructions.

Cortical ROS generation was measured concurrently with respirometry in the same cortical samples using methods previously described [4,5,6,12]. The concentration of protein carbonyl groups was measured in the additional left cortical samples, as previously described [5]. Protein carbonylation occurs when protein side chains are oxidized. A rise in the concentration of protein carbonyl groups is used as a marker of oxidative stress [6].

### 4.3. Statistical Analysis

The sample size was based on the resource equation using 10 to 20 degrees of freedom to meet significance. Statistical analysis was carried out using GraphPad Prism version 9.1.0 (GraphPad Software Inc., San Diego, CA, USA). All of the values were plotted as mean and standard error of the mean or standard deviation. The categorical variables were summarized using frequency and percentages. Continuous variables were summarized using mean and standard deviation or median and range if not normally distributed. Normality was assessed using the Shapiro–Wilk normality test. Differences among groups were compared using either Student’s *t*-test, one-way ANOVA, or non-parametric Wilcoxon rank-sum test or Kruskal−Wallis test, respectively, when the groups were not normally distributed. Differences were considered statistically significant when *p* < 0.05.

## 5. Conclusions

Controlled oxygenation with PaO_2_ < 150 mmHg during CPB does not significantly decrease acute cortical mitochondrial function or downstream indicators of oxidative injury and bioenergetic failure in acyanotic neonatal swine. We aim to expand upon this study by (1) investigating the oxygenation strategy in cyanotic animals and (2) using a survival model of CPB to better evaluate the short and long-term effects of oxygen level titration during pediatric CPB on the developing brain.

## Figures and Tables

**Table 1:** Animal characteristics, hemodynamics, and arterial blood gas variables at baseline.

Variable Mean (SD) or Median (Range)	Normoxia	Hyperoxia	*p*-Value
Weight (kg)	4.2 (0.2)	4.3 (0.5)	0.49
Age (days)	12 (11–12)	11 (11–12)	0.52
Heart rate (bpm)	117.4 (5.2)	108.6 (15.9)	0.28
MAP (mmHg)	59.6 (7.0)	67.3 (16.1)	0.36
Arterial pH	7.44 (0.07)	7.45 (0.04)	0.87
PaCO_2_ (mmHg)	43.6 (11.8)	43.8 (3.0)	0.97
PaO_2_ (mmHg)	82.8 (47.0)	81.0 (10.8)	0.94
Glucose (mg/dL)	73.6 (40.9)	84.6 (14.3)	0.59
Hematocrit (%)	26.0 (23–26)	23 (20–25)	0.06
Lactate (mmol/L)	1.3 (0.2)	1.4 (0.1)	0.21
Base Excess (mmol/L)	4.5 (3.0)	6.0 (2.5)	0.40

**Table 2:** PaO_2_ (mmHg) at baseline and throughout CPB, mean (SD) or median (range).

Timepoint	Normoxia	Hyperoxia	*p*-Value
Baseline	82.8 (47.0)	81.0 (10.8)	0.9
CPB 1st hour	87 (49–211)	338.5 (180–405)	**<0.001**
CPB 2nd hour	92.5 (80–159)	343 (270–499)	**<0.001**
CPB 3rd hour	95 (62–122)	363 (194–455)	**<0.001**

**Table 3:** Hemodynamic and arterial blood gas variables during CPB.

Variable, Mean (SD), or Median (Range)	Normoxia	Hyperoxia	*p*-Value
CPB 1st hour			
Heart rate (bpm)	134 (26)	150 (24)	0.4
MAP (mmHg)	66.5 (4.7)	60.6 (13.8)	0.4
Arterial pH	7.5 (0.07)	7.5 (0.03)	0.2
**PaO_2_ (mmHg)**	87 (49–211)	338.5 (180–405)	**<0.001**
**PaCO_2_ (mmHg)**	43.0 (6.9)	39.6 (2.9)	0.3
Glucose (mg/dL)	152.6 (57.6)	152.2 (47.0)	1.0
Hematocrit (%)	34.0 (2.4)	36.6 (2.3)	0.1
Lactate (mmol/L)	1.9 (0.4)	2.4 (1.2)	0.4
Base Excess (mmol/L)	7.4 (2.6)	9.4 (2.2)	0.2
CPB 2nd hour			
Heart rate	163 (8)	148 (26)	0.3
MAP	60.0 (5.7)	58.1 (4.8)	0.59
Arterial pH	7.5 (7.4–7.5)	7.5 (7.4–7.5)	0.8
**PaO_2_**	92.5 (80–159)	343 (270–499)	**<0.001**
**PaCO_2_**	43.4 (6.1)	44.2 (5.8)	0.8
Glucose	118 (98–270)	166 (99–232)	0.6
Hematocrit	32.8 (2.2)	36.2 (1.6)	**0.02**
Lactate	1.9 (0.5)	2.4 (1.2)	0.5
Base Excess	7.6 (2.1)	9.0 (3.0)	0.4
CPB 3rd hour			
Heart rate	157 (12)	117 (28)	**0.02**
MAP	63.1 (8.0)	56.0 (3.1)	0.1
Arterial pH	7.5 (0.09)	7.5 (0.05)	0.7
**PaO_2_**	95 (62–122)	363 (194–455)	**<0.001**
**PaCO_2_**	36 (30–67)	38 (37–45)	0.5
Glucose	157.4 (44.5)	155.6 (42.4)	1.0
Hematocrit	36 (6.6)	32.2 (1.6)	0.3
Lactate	2.3 (0.5)	2.6 (1.3)	0.6
Base Excess	7.3 (2.0)	8.7 (3.9)	0.5

## Data Availability

The data presented in this study are available upon request from the corresponding author.

## Supplementary Materials

The supporting information can be downloaded at: https://www.mdpi.com/article/10.3390/ijms25105466/s1. References [4,5,6,12,14,16] are cited in the supplementary materials.
