# Supplementary material for: Normoxic Management during Cardiopulmonary Bypass Does Not Reduce Cerebral Mitochondrial Dysfunction in Neonatal Swine
Source: Int J Mol Sci. 2024 May 17;25(10):5466. doi: 10.3390/ijms25105466 (PMC11122014; doi:10.3390/ijms25105466)
Supplement: Supplementary file 1 [file ijms-25-05466-s001.zip › ijms-2967458-supplementary.pdf]

Supplemental Material

Perioperative preparation

Animals were sedated with intramuscular ketamine (20mg/kg), followed by inhaled isoflurane in 100% fraction of inspired oxygen (FiO_2) via a snout mask. After endotracheal intubation, they were ventilated with tidal volumes of 10mL/kg and positive end-expiratory pressure of 5cm H_20. Minute ventilation was titrated to an end-tidal carbon dioxide ($EtCO_2$) of 38-42 mmHg and FiO_2 was weaned to 21%. Animals were positioned prone on a warming blanket with the following continuous monitors: pulse oximetry, electrocardiogram, and temperature (rectal and nasal). Body temperature was maintained at 38-40°C before CPB.

A microdialysis catheter was inserted 0.5cm deep into the brain parenchyma at the grey-white junction of the right frontal cortex via a 2mm frontal burr hole (CMA 71 Elite, mDialysis, Sweden, Figure 1). Sterile saline was infused at 1µL/min and cerebral microdialysate (i.e., interstitial fluid as a representation of cerebral extracellular fluid) was sampled every 20-40 minutes from the time of catheter placement through euthanasia and stored at -80°C. The concentrations of glycerol, lactate, and pyruvate were measured in each microdialysate sample by a blinded technician using the automated ISCUS FlexTM Microdialysis Analyzer (mDialysis).

The left femoral artery and vein were instrumented for continuous arterial pressure monitoring and central venous access. Whole blood samples were collected at baseline and every 1 hour during CPB for serial blood gas and activated clotting time (ACT) measurements. Intravenous infusions of fentanyl (25-200µg/kg/min) and dexmedetomidine (0.5-2µg/kg/min) were initiated and isoflurane was titrated to 1-1.5%.

Operative details and CPB parameters

After systemic heparinization (4000IU), the right external jugular vein (10Fr venous cannula) and right carotid artery (8Fr arterial cannula) were cannulated for CPB (Figure 1). Once the ACT measured >450 seconds, CPB was initiated using circuitry and priming methods as those previously described. Target flow rate was >100mL/kg/minute and cooling commenced with a target temperature of 34°C, no faster than 1°C/minute. Target mean arterial pressure range on CPB was 45-65mmHg. Minute ventilation was reduced to tidal volume 10mL/kg and respiratory rate 10 breaths per minute during CPB. Sweep was titrated to a partial pressure of arterial carbon dioxide ($PaCO_2$) of 35-45mmHg. The FiO_2 was titrated to a PaO_2 >300mmHg for the hyperoxia group and PaO_2 <150mmHg for the normoxia group. Isoflurane via

the circuit was maintained at 1% and inhaled isoflurane was discontinued. Serum ACT was measured every 30 minutes and maintained at >450 seconds with supplemental doses of heparin. Hematocrit was maintained at >28% using swine donor blood. Alpha-stat blood gas management was used while at mild hypothermia.

Euthanasia and terminal biospecimen collection

Necropsy:

After 3 hours of CPB, euthanasia was induced with high-dose intravenous potassium chloride followed by immediate craniectomy and whole brain excision. Cortical slices of the left hemisphere were isolated and placed into an ice-cold buffer (320mM sucrose, 2mM EGTA, 10mM Trizma base, pH 7.4). These fresh tissue samples were homogenized and prepared for ex-vivo high-resolution mitochondrial respirometry, and quantification of citrate synthase activity as previously described.

High-resolution mitochondrial respirometry, quantification of ROS generation, and mitochondrial content:

Mitochondrial respiration in left cortex homogenates (1mg/mL) was measured using a highresolution respirometer (Oxygraph-2k; OROBOROS Instruments, Austria) and a substrate–uncoupler– inhibitor titration (SUIT) protocol as previously described.[11,12,13,17] This protocol facilitates the transfer of electrons and consumption of oxygen by each of the four mitochondrial complexes in the electron transport chain. The following respiratory states were measured: maximal oxidative phosphorylation via complex I (OXPHOS CI), maximal convergent oxidative phosphorylation via complexes I and II (OXPHOS CI+CII), oligomycin-induced proton cycling through mitochondria without ATP synthesis (LEAK), maximal convergent non-phosphorylating respiration of the electron transport system via complexes I and II (ETS CI+CII), and via complex II alone (ETS CII), and complex IV activity.Quantified values for each of these respiratory states were compared per milligram of tissue and Normali zed to the concentration of citrate synthase in each homogenate sample. Citrate synthase is a mitochondrial enzyme routinely used as a surrogate of mitochondrial content.[18] Citrate synthase activity (nmol/mg/min) in the same cortical homogenates used for respirometry was measured with a commercially available kit (Citrate Synthase Assay Kit, CS0720, Sigma-Aldrich, St Louis, MO) according to the manufacturer's instructions.

Measurement of cortical ROS and cortical protein carbonylation:

Dysfunctional mitochondria generate significant amounts of ROS which contribute to downstream redox signaling, oxidative damage to cellular proteins and lipids, and subsequent secondary brain injury. Cortical ROS generation was measured in the same cortical samples used for respirometry with the O2k-Fluorescence LED2-Module (Oxygraph-2k) as previously described.[11,12,13,17]
Protein carbonylation is one type of protein modification that occurs when protein side chains are oxidized. A rise in the concentration of protein carbonyl groups has been used as a marker of oxidative stress.[19] Frozen left cortex samples (50 mg/mL) were homogenized in PBS, pH 7.4, containing protease and phosphatase inhibitor and BHT. Homogenates were centrifuged at 16,000g for 10 minutes at 4°C and the supernatant was incubated with streptomycin 1% for 30 min at 4°C to remove nucleic acids. Samples were centrifuged again at 16,000g for 10 minutes at 4°C and supernatant was collected and the concentration of protein carbonyl groups was measured using a commercially available ELISA kit (OxiSelect™ Protein Carbonyl ELISA Kit #STA-310, San Diego, CA) according to the manufacturer's Instructions.

The large animal model of cardiopulmonary bypass described here has been used in previous studies reported in the literature.[1-4] The molecular and histopathological methods described here have been used in several studies from our lab published in the literature.[1,2,4-6]

References:

1. Mavroudis CD, Karlsson M, Ko T, et al. Cerebral mitochondrial dysfunction associated with deep hypothermic circulatory arrest in neonatal swine. *Eur J Cardiothorac Surg*. 2018;54(1):162-168.
2. Volk LE, Mavroudis CD, Ko T, et al. Increased cerebral mitochondrial dysfunction and reactive oxygen species with cardiopulmonary bypass. *Eur J Cardiothorac Surg*. 2021;59(6):1256-1264.
3. Myung RJ, Kirshbom PM, Petko M, et al. Modified ultrafiltration may not improve neurologic outcome following deep hypothermic circulatory arrest. *Eur J of Cardiothoracic Surg*. **2003**;24(2):243-248.
4. Aronowitz DI, Geoffrion TR, Piel S, et al. Early Impairment of Cerebral Bioenergetics After Cardiopulmonary Bypass in Neonatal Swine. *World J for Pediatr Cong*. 2024; in print.
5. Marquez AM, Morgan RW, Ko T, et al. Oxygen Exposure During Cardiopulmonary Resuscitation Is Associated With Cerebral Oxidative Injury in a Randomized, Blinded, Controlled, Preclinical Trial. *J Am Heart Assoc*. 2020;9(9):e015032.
6. Kilbaugh TJ, Bhandare S, Lorom DH, et al. Cyclosporin A preserves mitochondrial function after traumatic brain injury in the immature rat and piglet. *J Neurotrauma*. 2011;28(5):763-774.
